# Supplementary material for: Long-Time Dynamics of Selected Molecular-Motor Components Using a Physics-Based Coarse-Grained Approach
Source: Biomolecules. 2023 Jun 5;13(6):941. doi: 10.3390/biom13060941 (PMC10296118; doi:10.3390/biom13060941)
Supplement: Supplementary file 1 [file biomolecules-13-00941-s001.zip › biomolecules-2409539-supplementary.pdf]

Supplementary Material for

Long-Time Dynamics of Selected Molecular-Motor Components by Using a Physics-Based Coarse-Grained Approach

A. Liwo, M. Pyrka, C. Czaplewski, X. Peng, A. J. Niemi

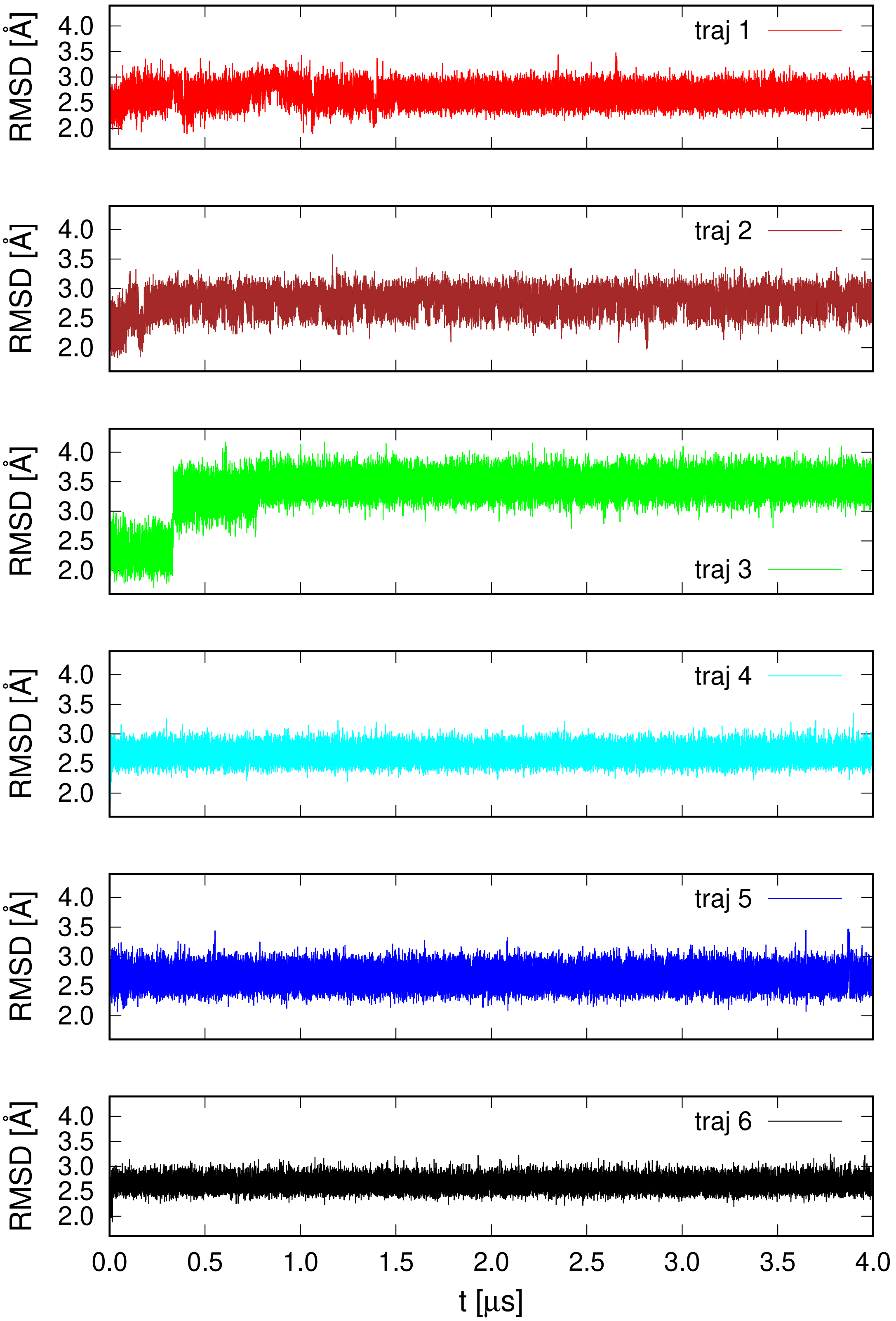

Figure S1: Variation of C^{α}-RMSD with simulation time for the 6 microcanonical MD trajectories of the 4YY2 system.

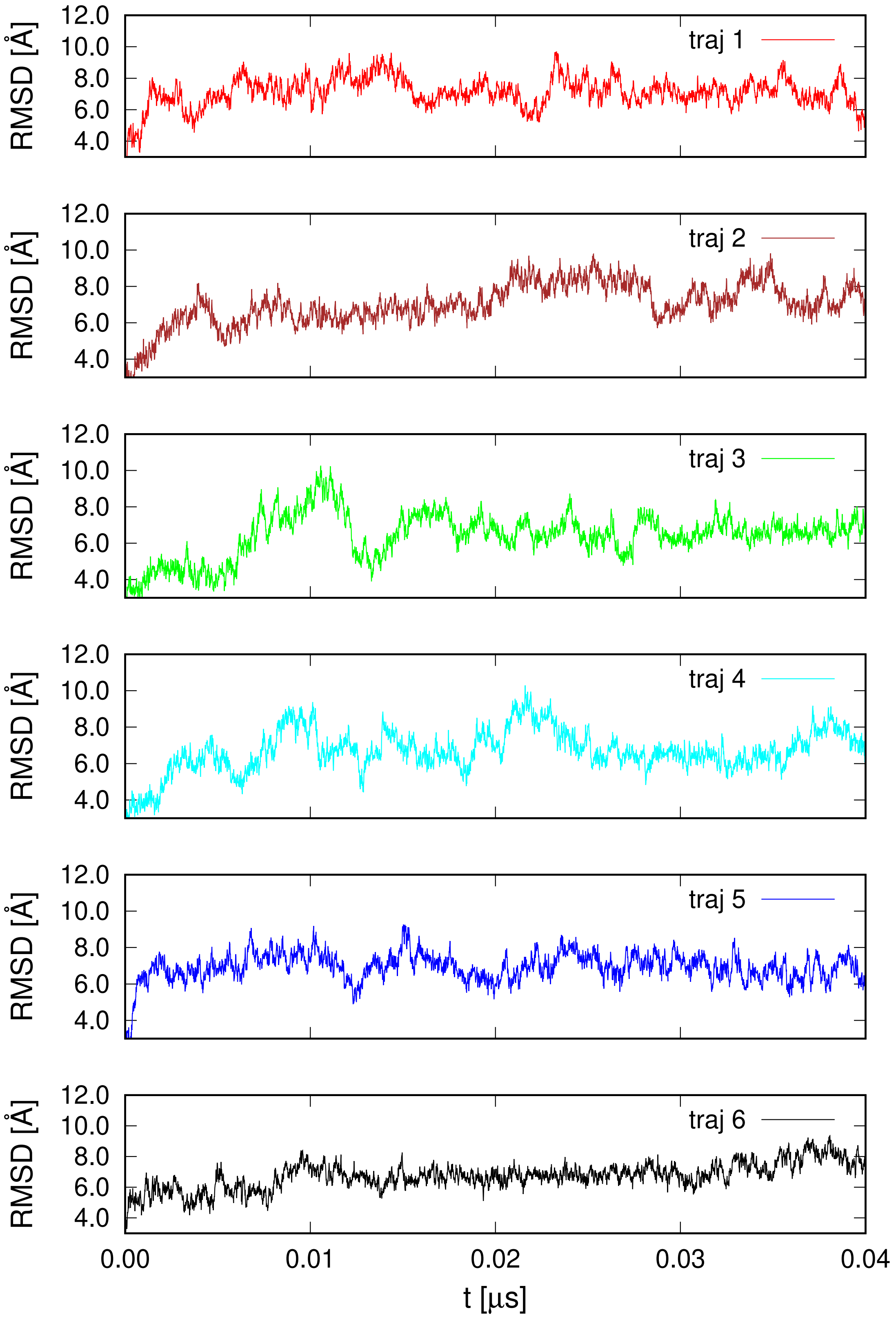

Figure S2: Variation of C^{α}-RMSD with simulation time for the 6 canonical MD trajectories of the 4YY2 system.

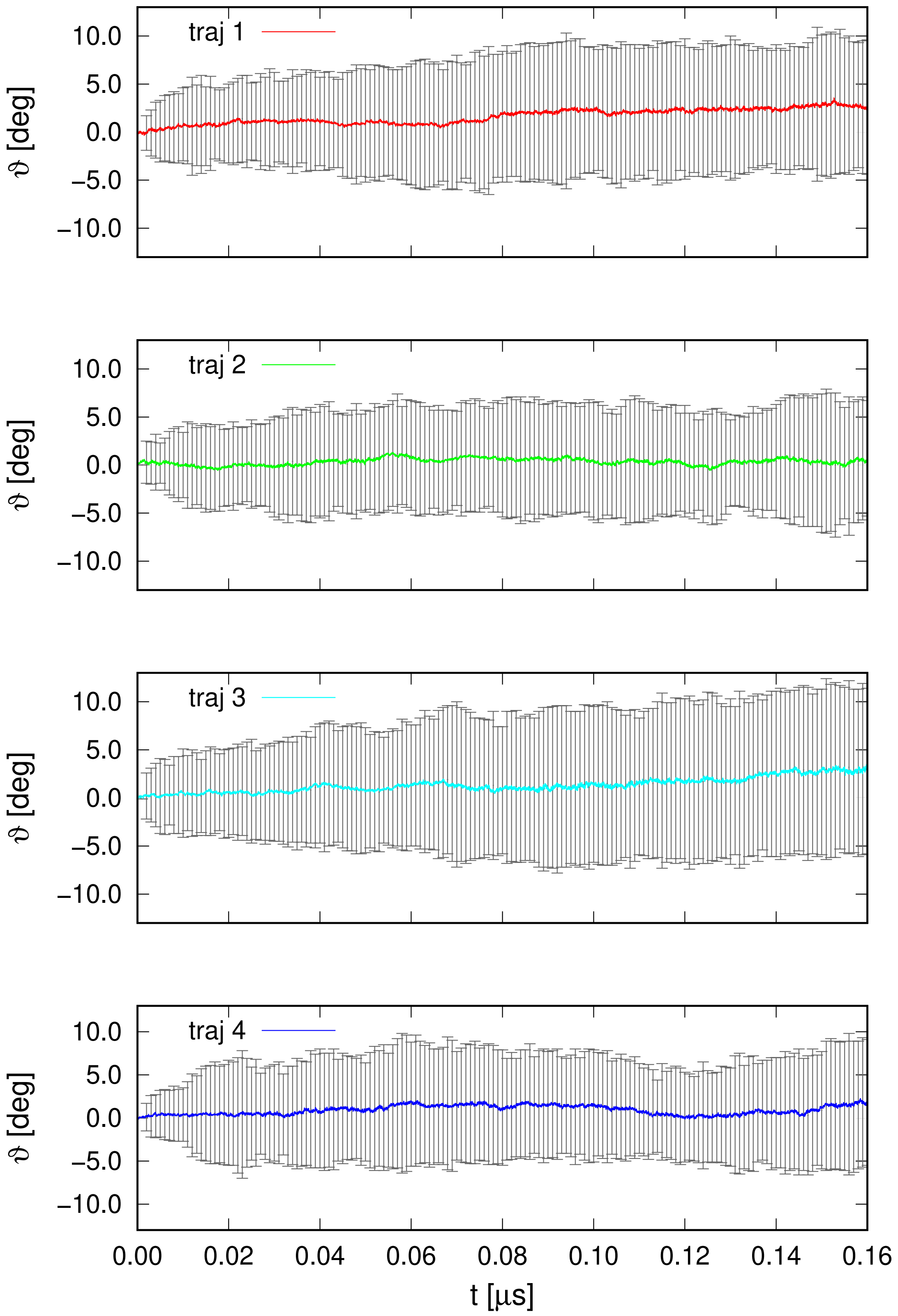

Figure S3: Variation of the average rotation angle ϑ (Equation (6) of the main text) with simulation time for the 4 microcanonical MD trajectories with type I restraints of the 6SD5 system. The errorbars (gray) amount to $\pm$ standard deviation and have been drawn every 100th point to avoid overcrowding the plot.

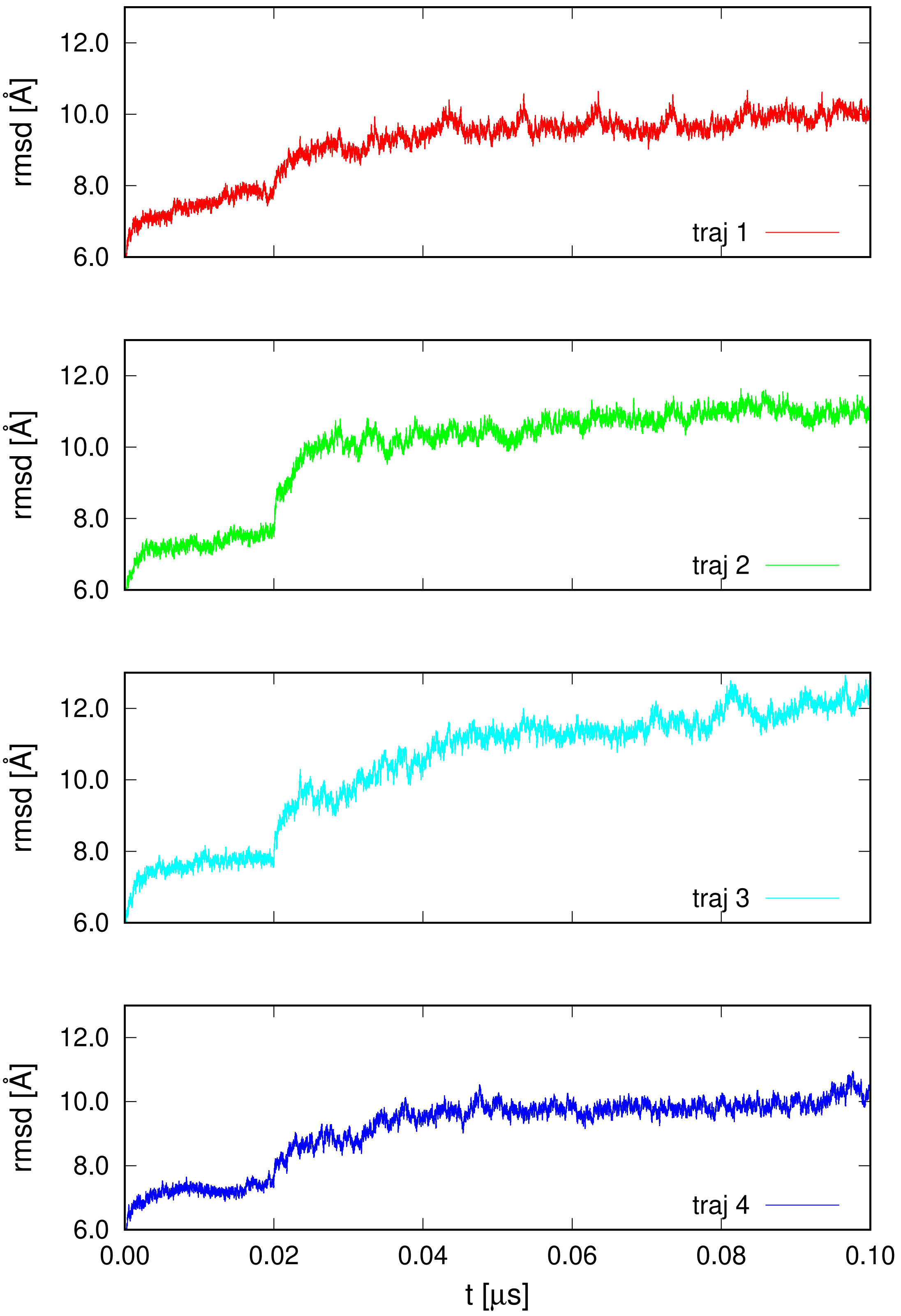

Figure S4: Variation of C^{α}-RMSD with simulation time for the 4 canonical MD trajectories with type I restraints of the 6SD5 system.

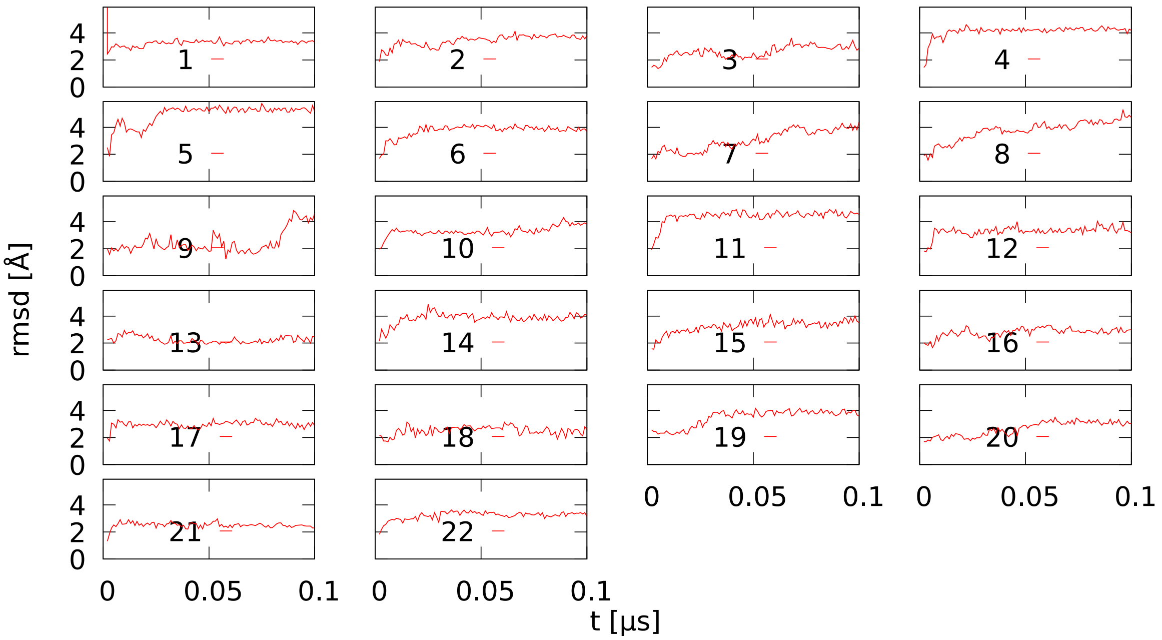
rmsd [Å]
t [μs]
1
2
3
4
5
6
7
8
9
10
11
12
13
14
15
16
17
18
19
20
21
22
0
0.05
0.1

A

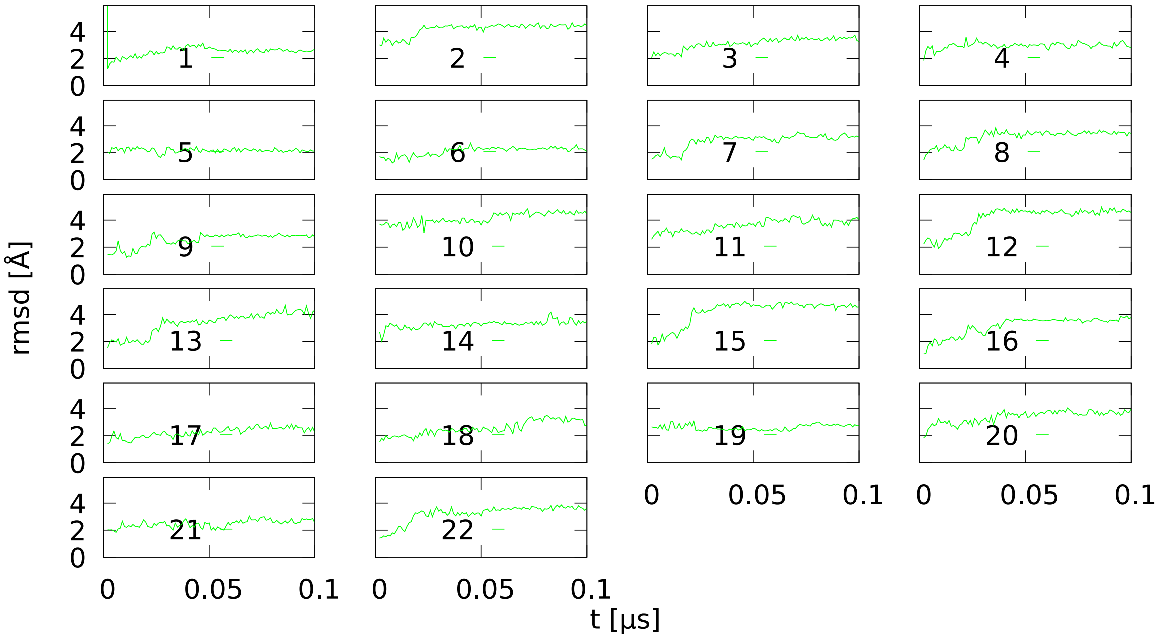
rmsd [Å]
t [μs]
1
2
3
4
5
6
7
8
9
10
11
12
13
14
15
16
17
18
19
20
21
22
0
0.05
0.1

B

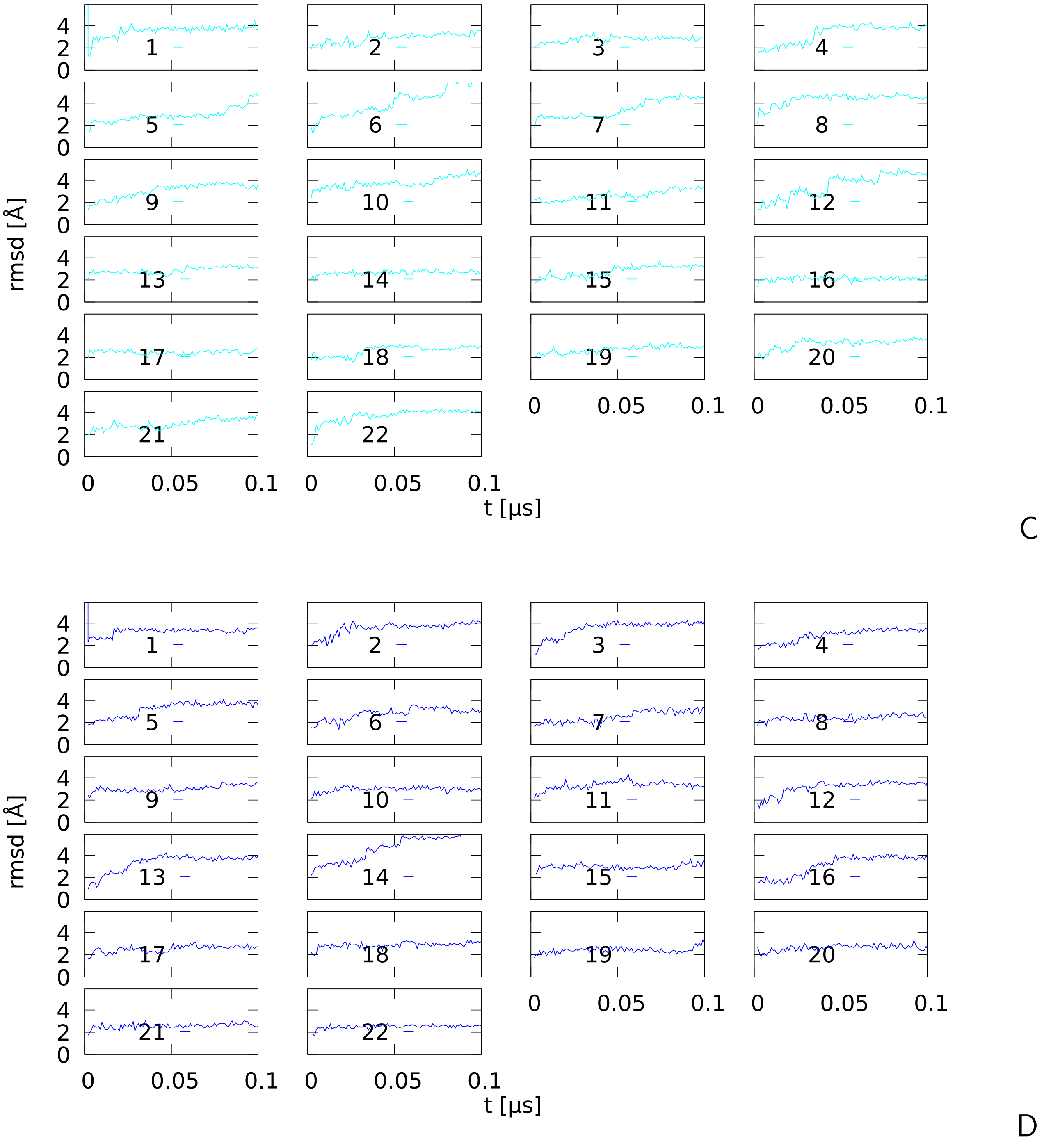

Figure S5: Variation of C^{α}-RMSD of the monoomers of 6SD5 from their initial structures with simulation time for the 4 canonical MD trajectories (A – D) with type I restraints. The sub-panels are labelled with the numbers of the consecutive monomers.

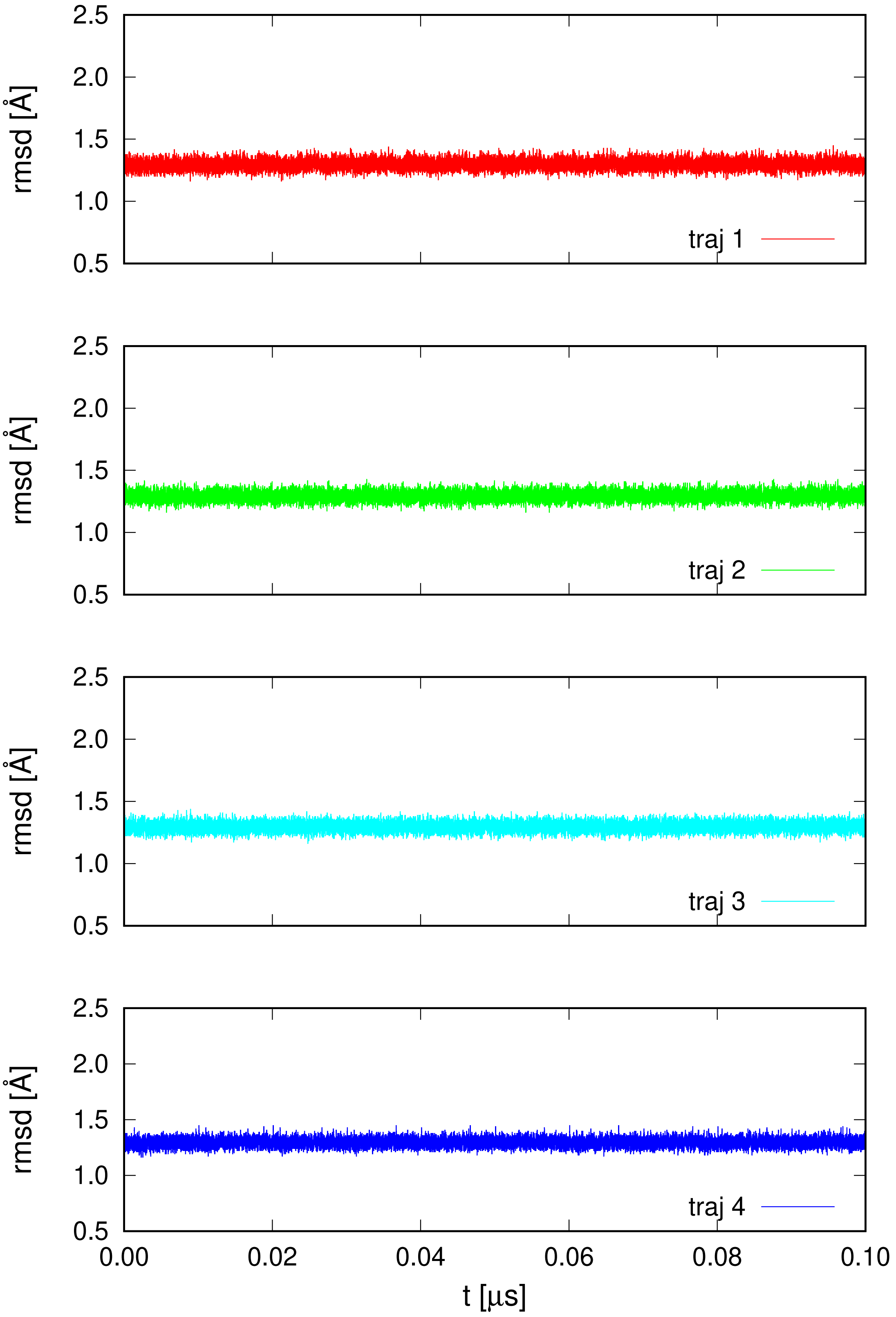

Figure S6: Variation of C^{α}-RMSD with simulation time for the 4 canonical MD trajectories with type II restraints of the 6SD5 system.

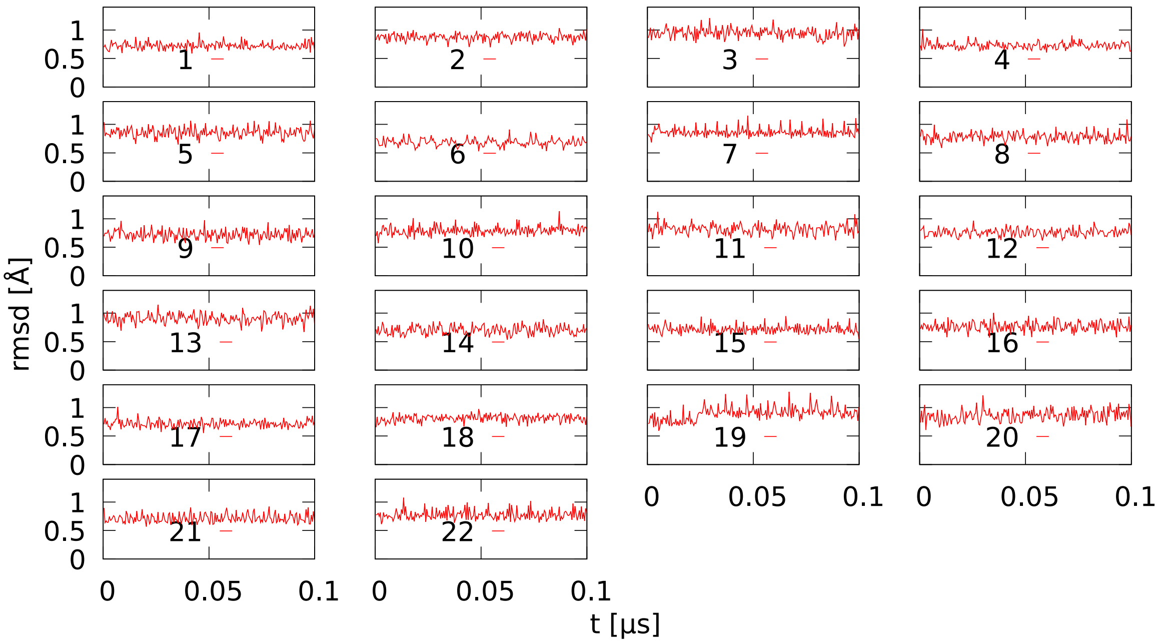
rmsd [Å]
t [μs]
0
0.5
1
0
0.05
0.1
1 2 3 4 5 6 7 8 9 10 11 12 13 14 15 16 17 18 19 20 21 22

A

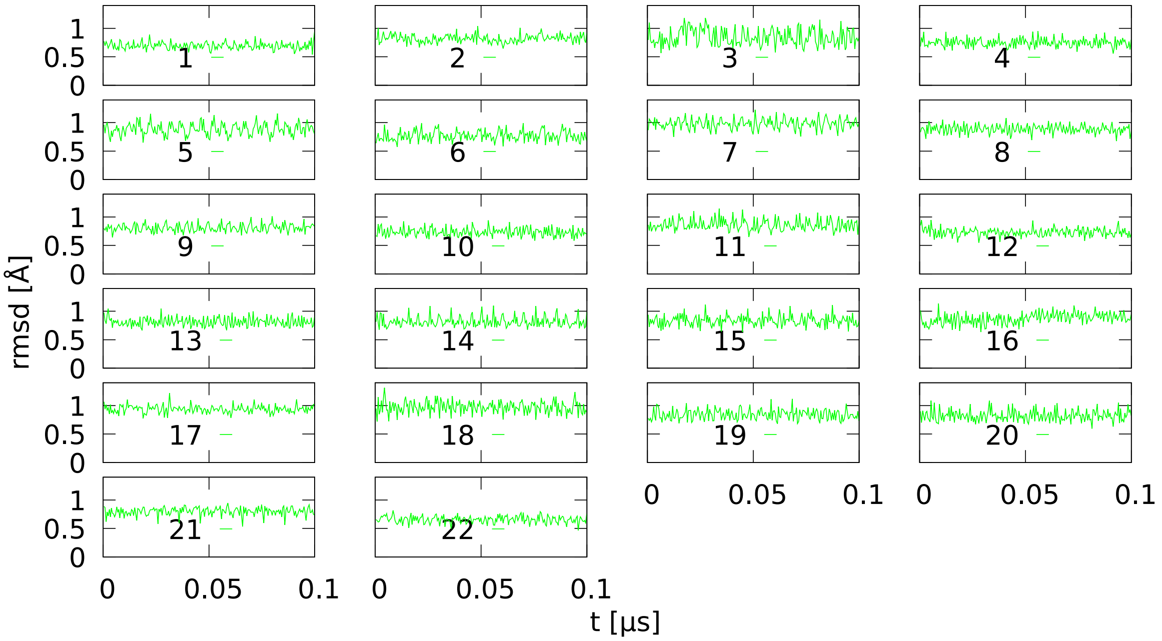
rmsd [Å]
t [μs]
0
0.5
1
0
0.05
0.1
1 2 3 4 5 6 7 8 9 10 11 12 13 14 15 16 17 18 19 20 21 22

B

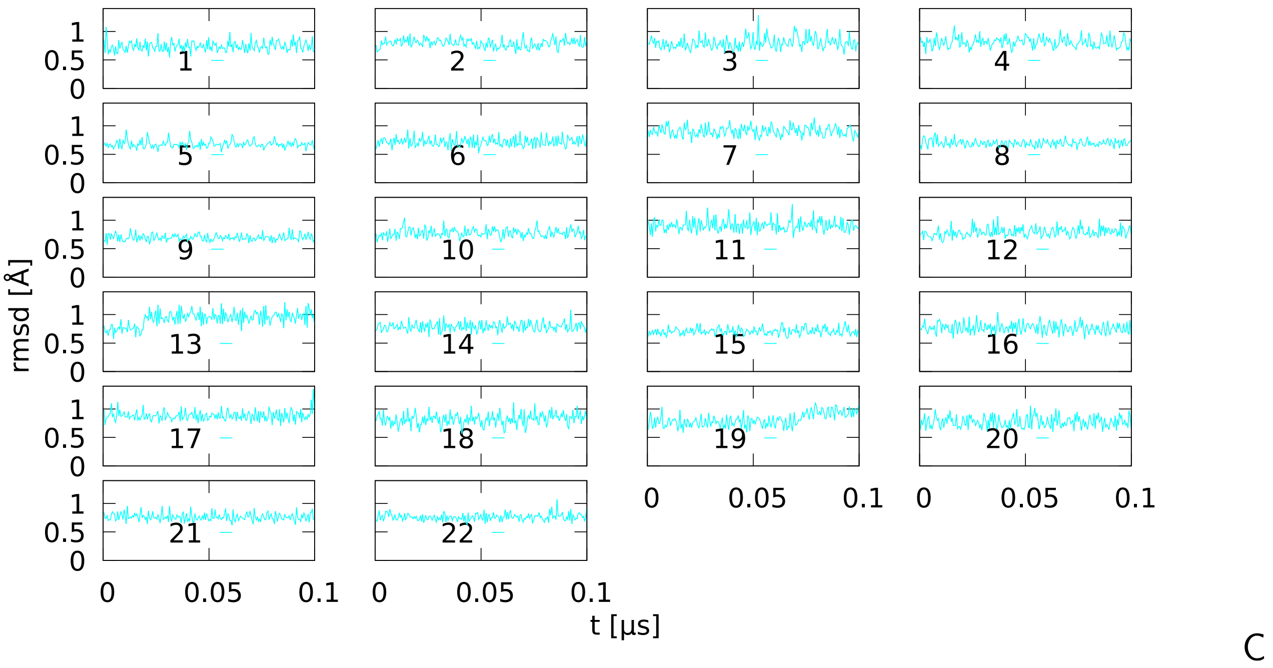

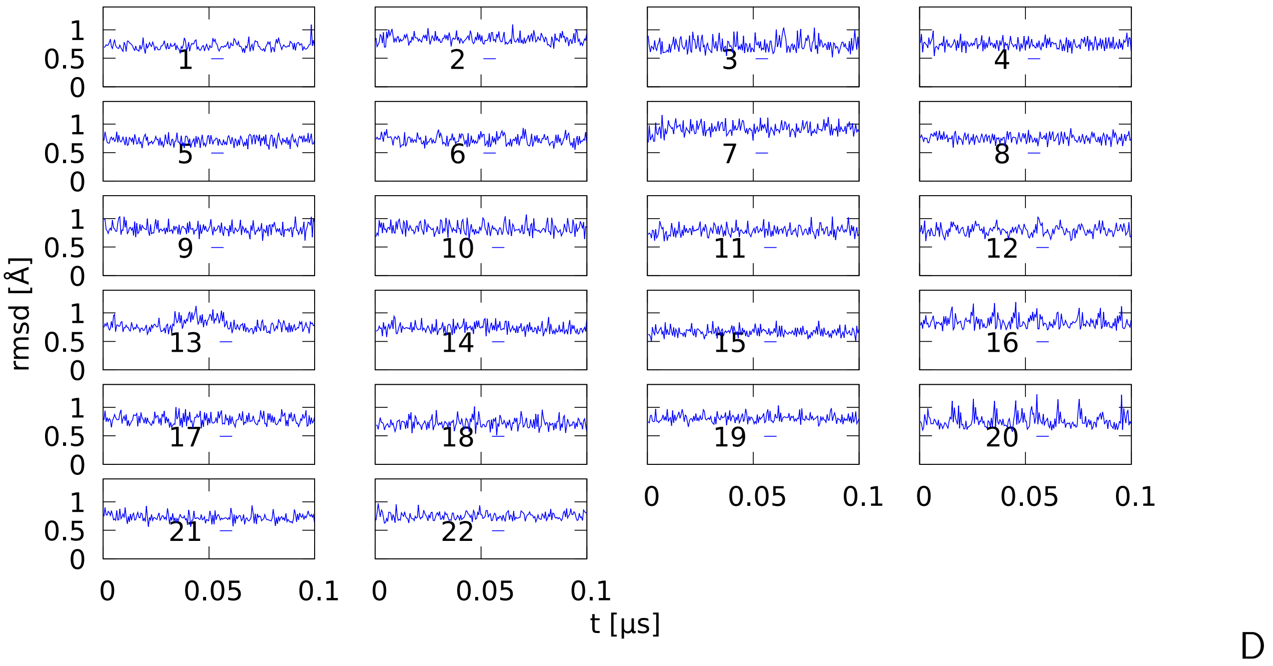

Figure S7: Variation of C^{α}-RMSD of the monoomers of 6SD5 from their initial structures with simulation time for the 4 canonical MD trajectories (A – D) with type II restraints. The sub-panels are labelled with the numbers of the consecutive monomers.

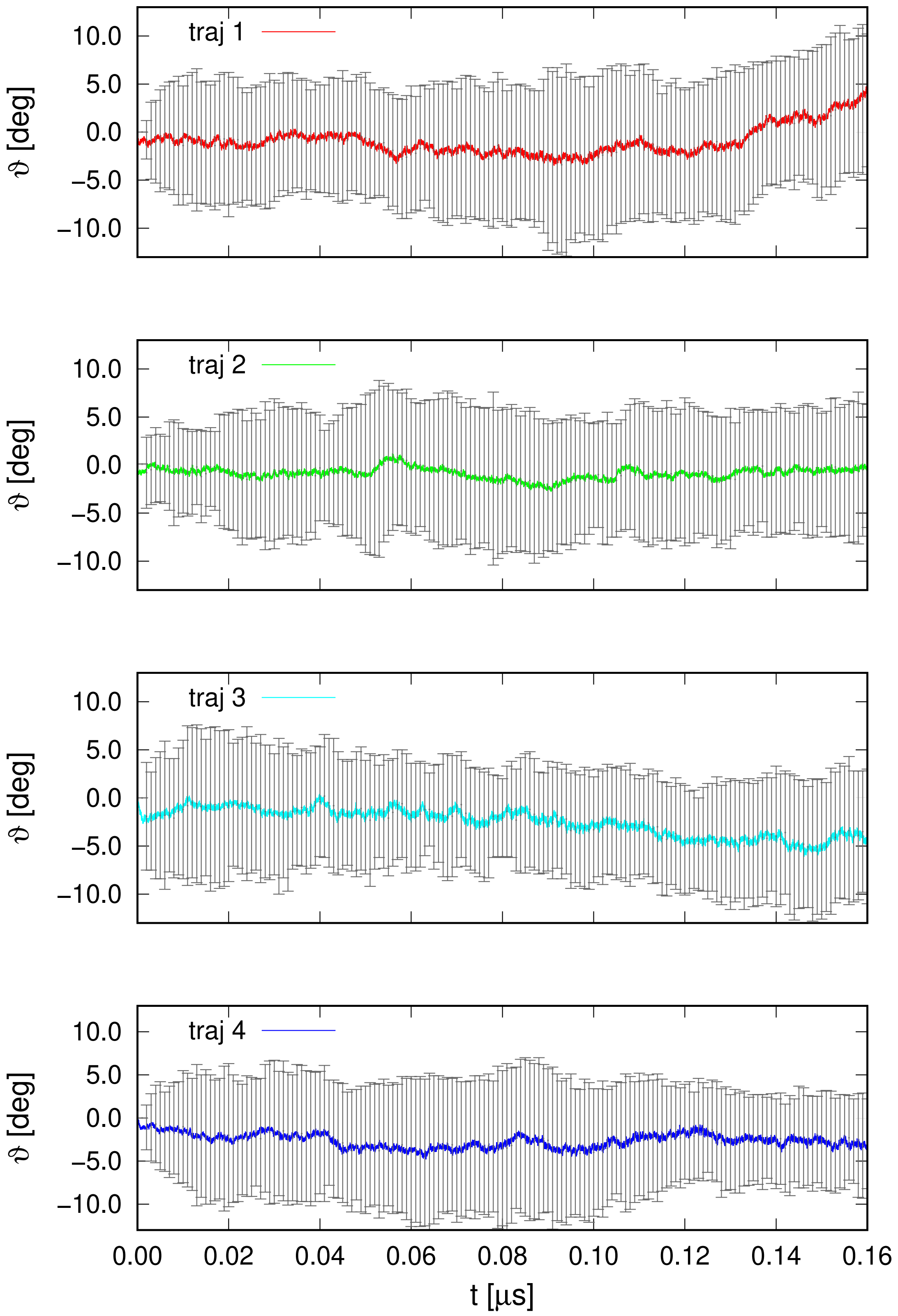

Figure S8: Variation of the average rotation angle ϑ (Equation (6) of the main text) with simulation time for the 4 microcanonical MD trajectories with type I restraints of the 2BL2 system. The errorbars (gray) amount to $\pm$ standard deviation and have been drawn every 100th point to avoid overcrowding the plot.

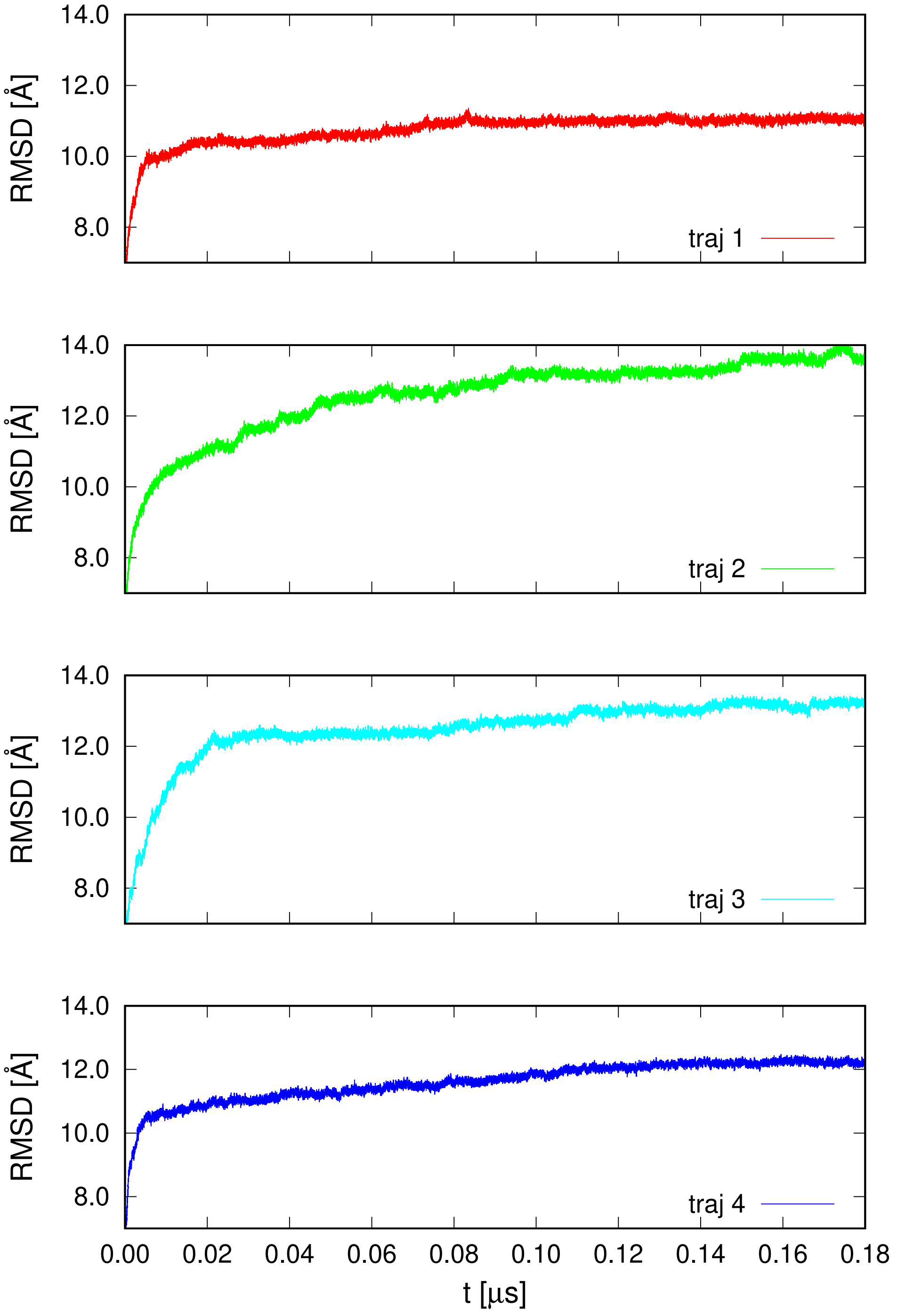

Figure S9: Variation of C^{α}-RMSD with simulation time for the 4 canonical MD trajectories with type I restraints of the 2BL2 system.

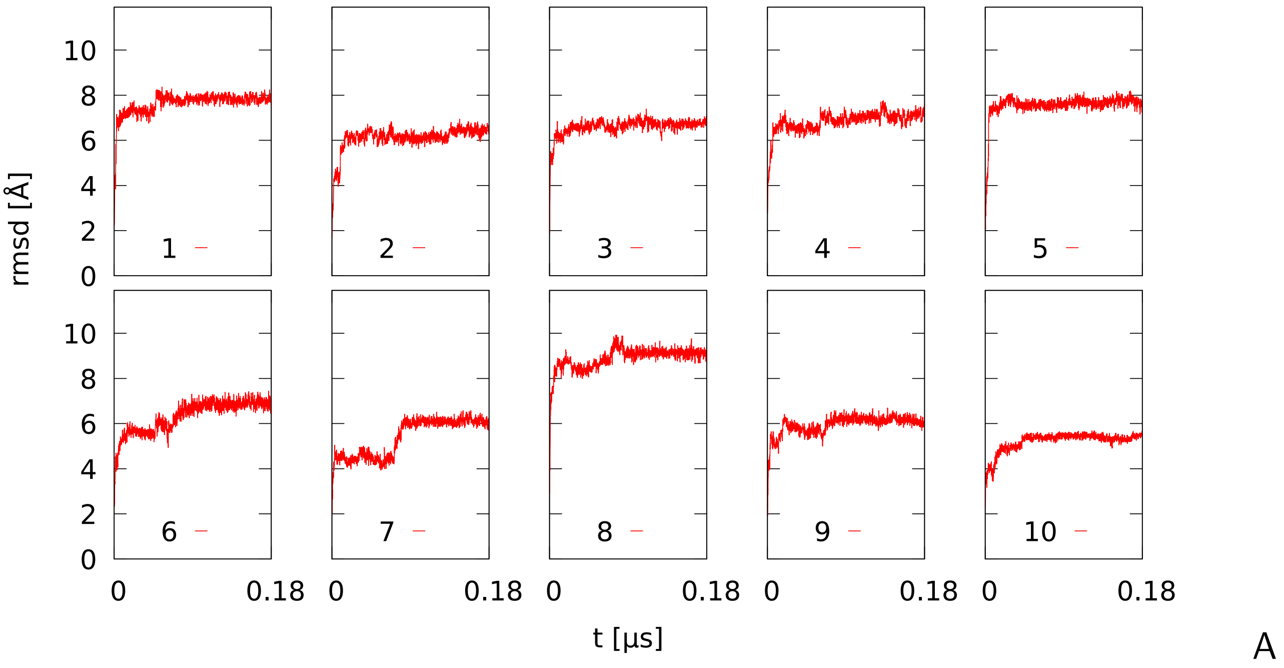
rmsd [Å]
10
8
6
4
2
0
1
2
3
4
5
6
7
8
9
10
0
0.18
t [μs]
A

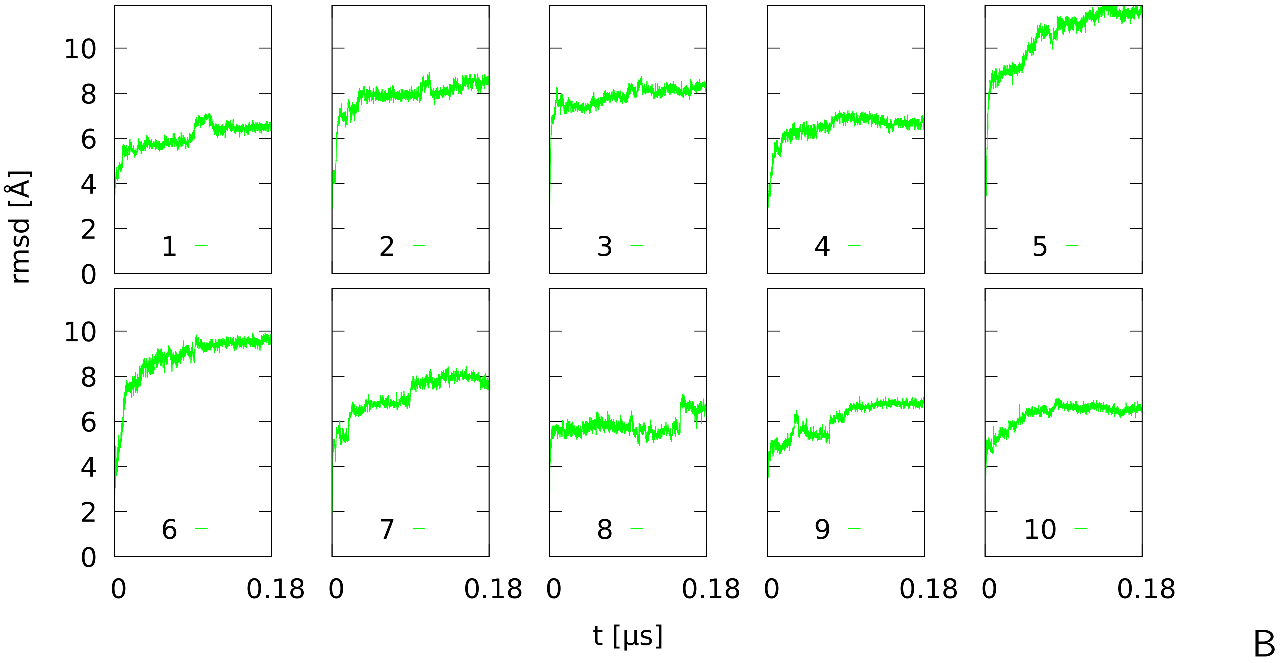
rmsd [Å]
10
8
6
4
2
0
1
2
3
4
5
6
7
8
9
10
0
0.18
t [μs]
B

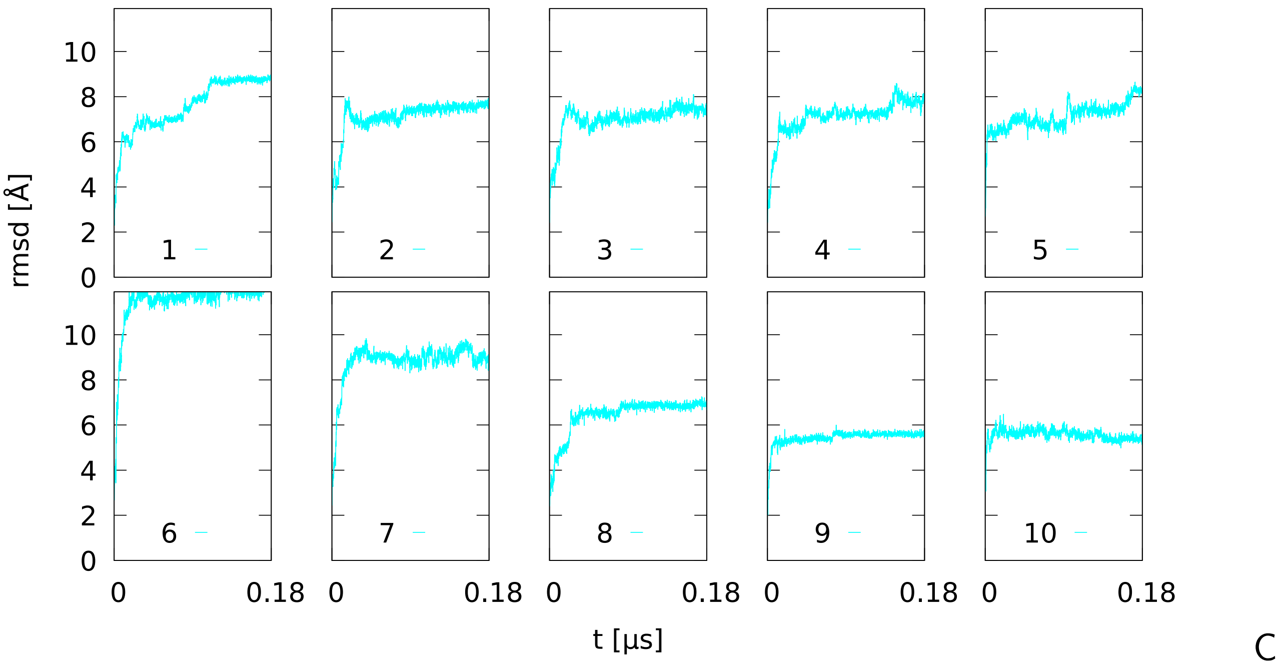

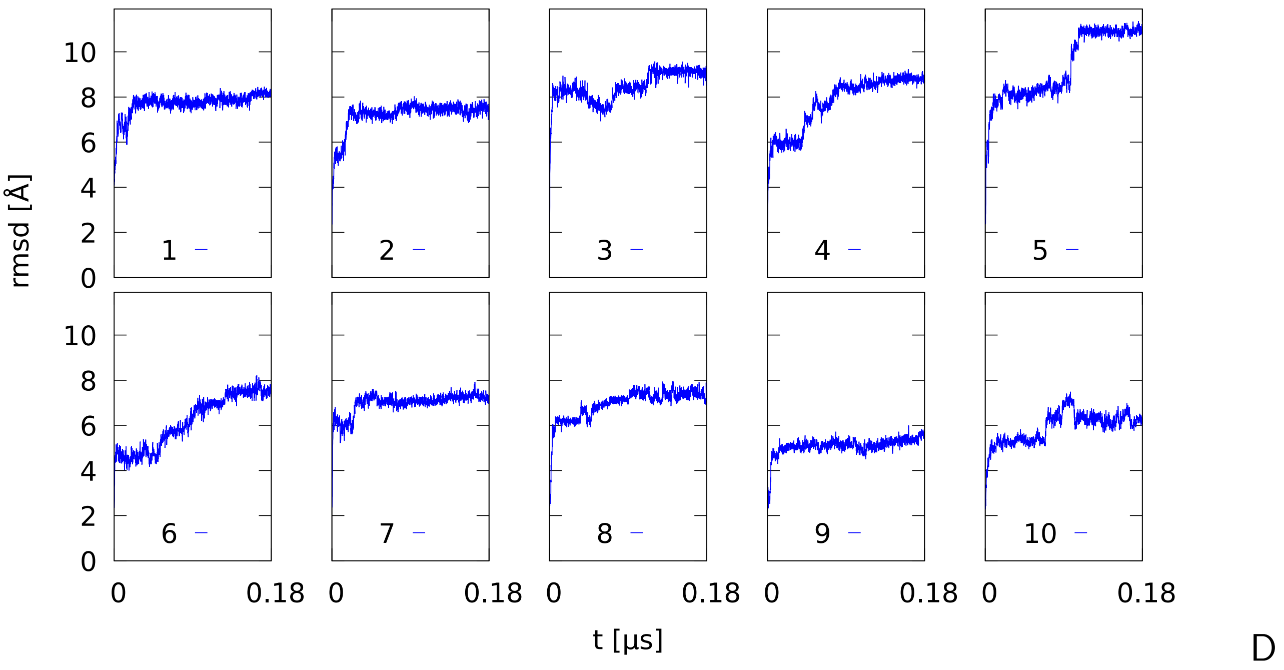

Figure S10: Variation of C^{α}-RMSD of the monoomers of 2BL2 from their initial structures with simulation time for the 4 canonical MD trajectories (A – D) with type I restraints. The sub-panels are labelled with the numbers of the consecutive monomers.

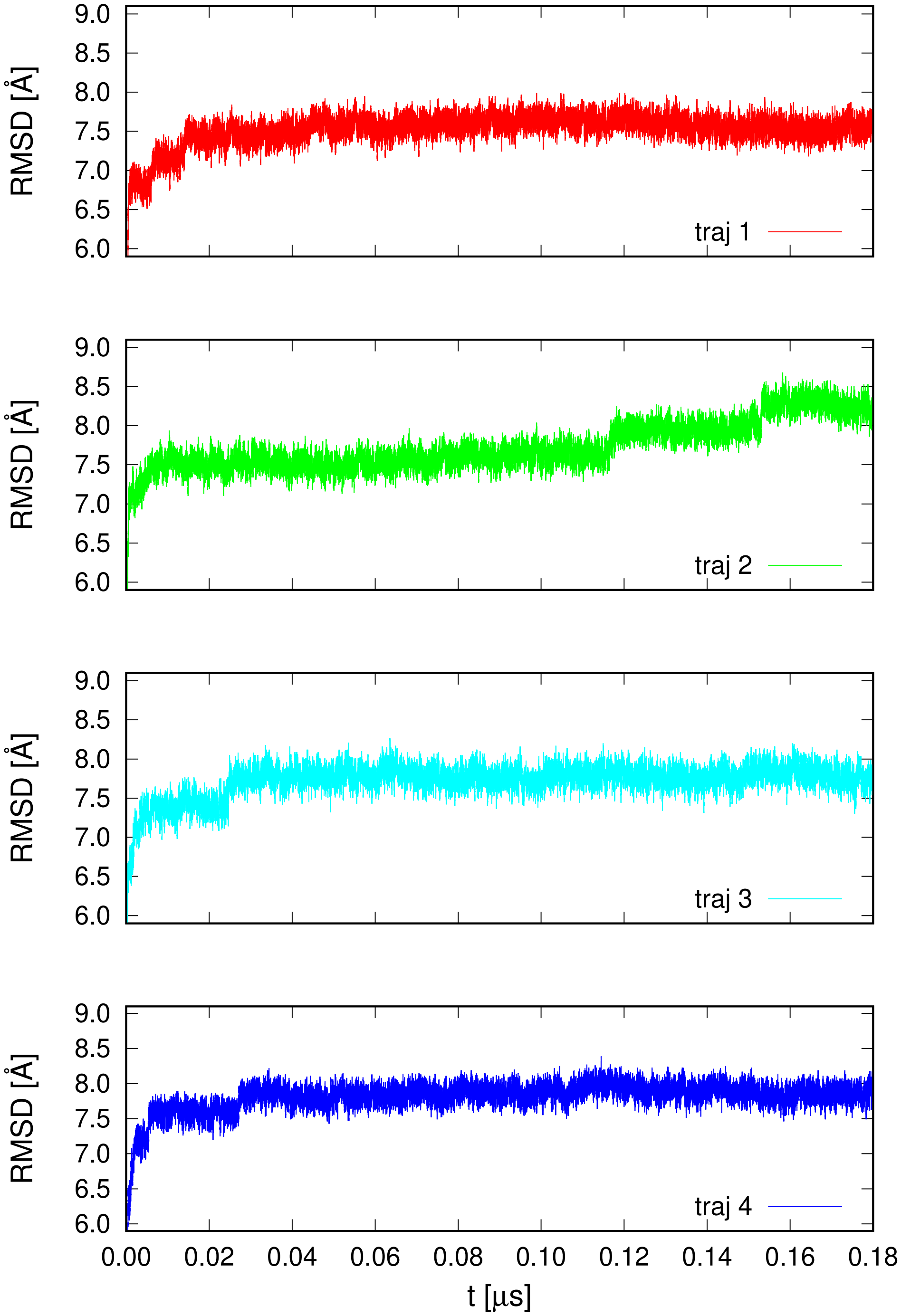

Figure S11: Variation of C^{α}-RMSD with simulation time for the 4 canonical MD trajectories with type II restraints of the 2BL2 system.

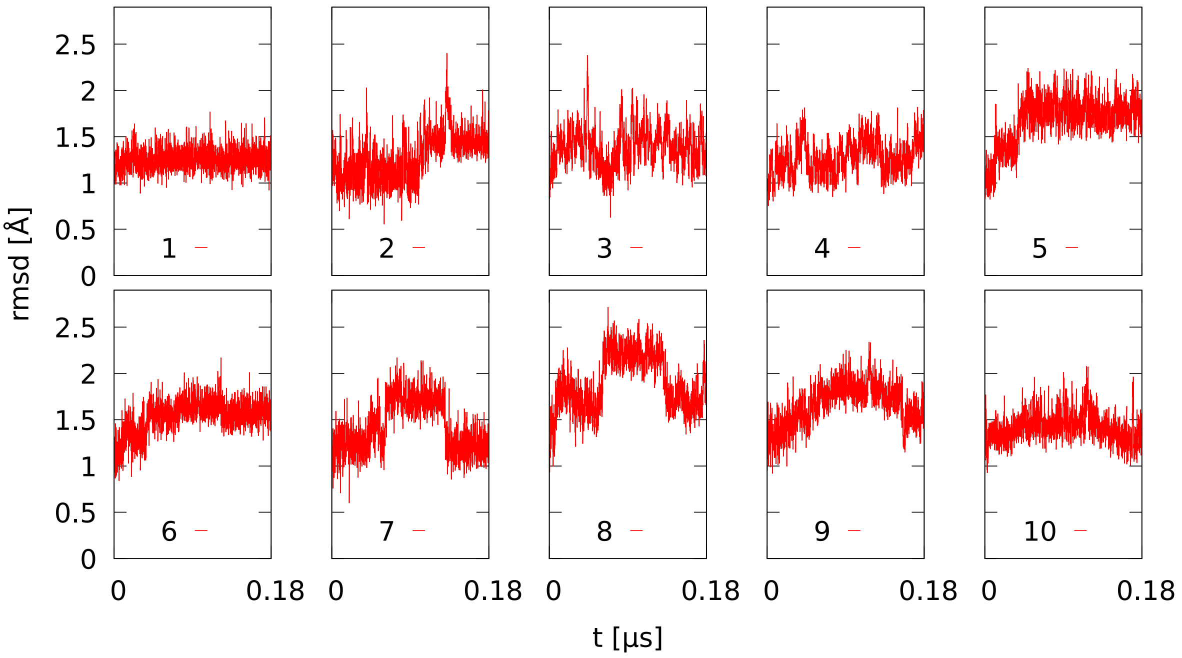

rmsd [Å]
2.5
2
1.5
1
0.5
0
1
2
3
4
5
6
7
8
9
10
0
0.18
t [µs]

A

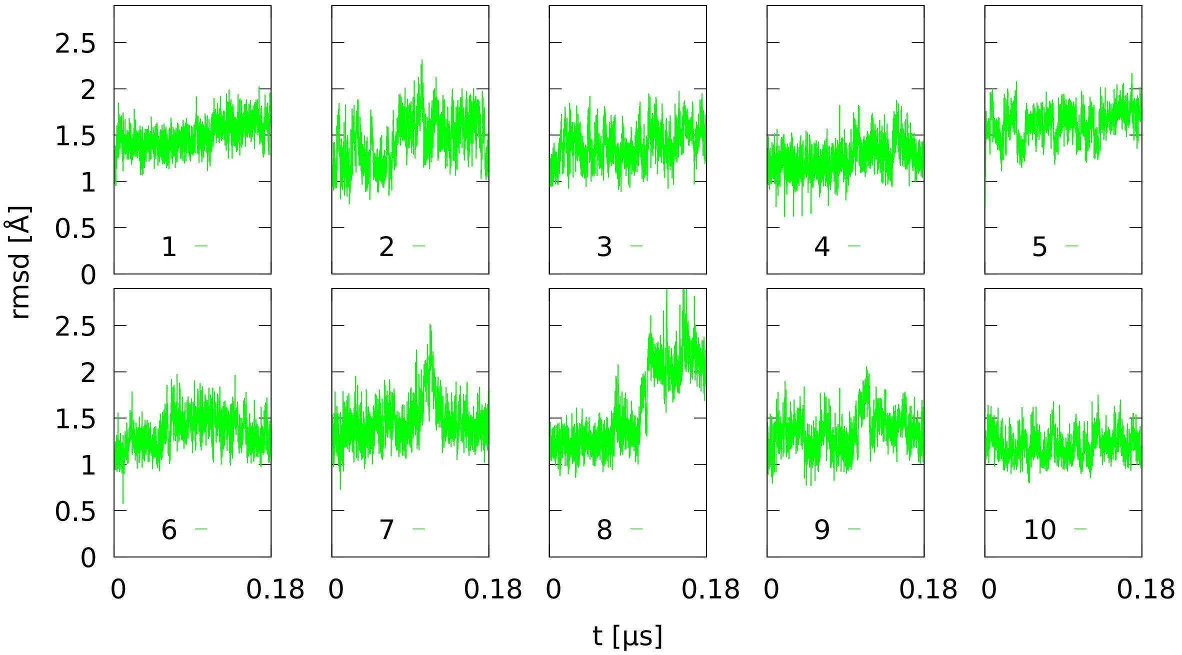

rmsd [Å]
2.5
2
1.5
1
0.5
0
1
2
3
4
5
6
7
8
9
10
0
0.18
t [µs]

B

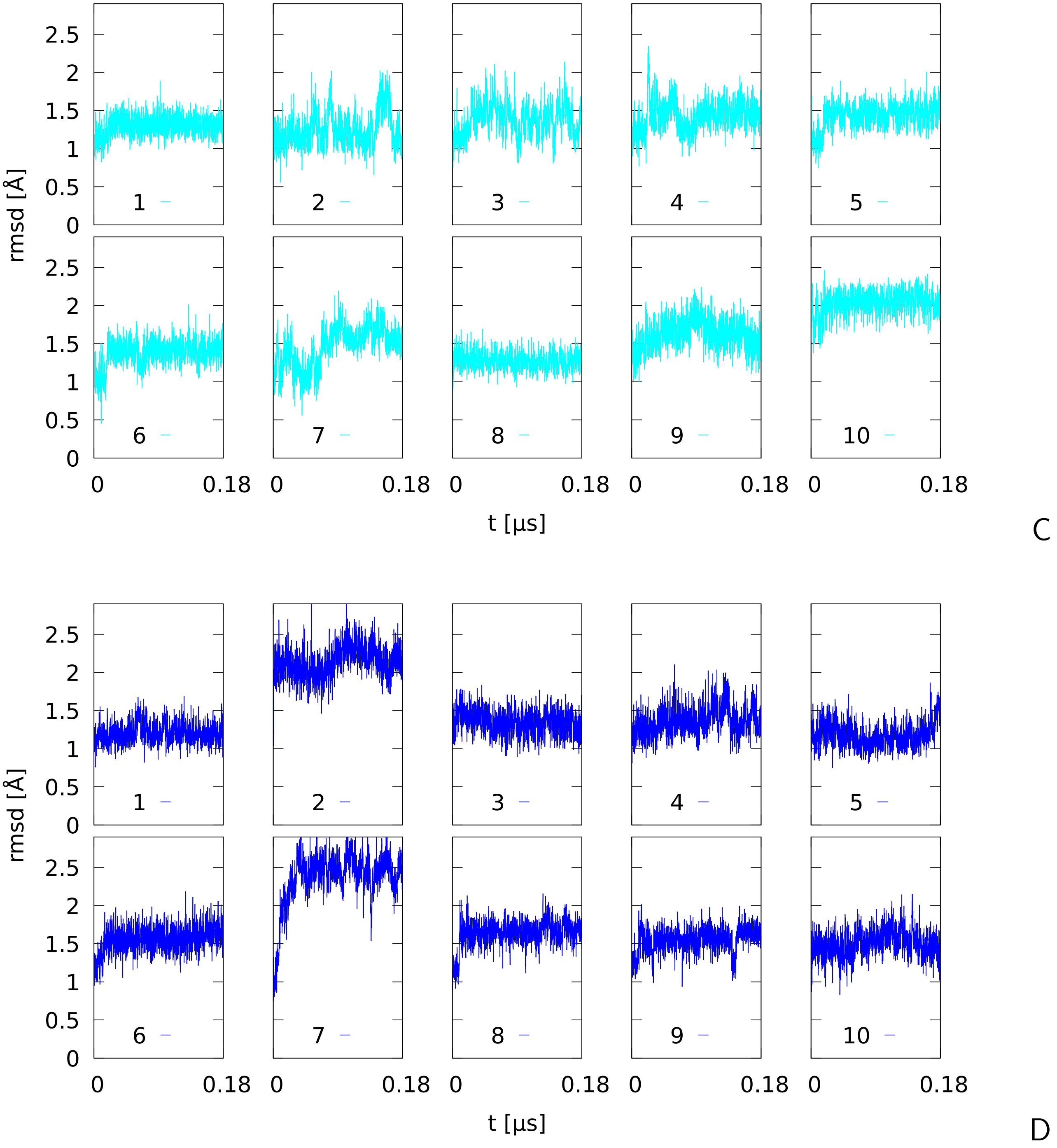

Figure S12: Variation of C^{α}-RMSD of the monoomers of 2BL2 from their initial structures with simulation time for the 4 canonical MD trajectories (A – D) with type II restraints. The sub-panels are labelled with the numbers of the consecutive monomers.
